# OLDER AMERICANS ACT: TARGETING OLDER ADULTS WITH GREATEST SOCIAL AND ECONOMIC NEEDS

**DOI:** 10.1093/geroni/igae098.0646

**Published:** 2024-12-31

**Authors:** Kristen Robinson

**Affiliations:** Administration for Community Living, Washington, District of Columbia, United States

## Abstract

The overall purpose of the Older Americans Act (OAA) is to help older adults remain in their communities through the provision of long-term services and supports. The Administration for Community Living administers the National Survey of OAA Participants (NSOAAP) to collect data on OAA clients in six service areas (home-delivered meals, congregate meals, homemaker services, transportation services, case management services, and caregiver support services). The annual survey includes questions on consumer assessment of service quality as well as client characteristics such as health status, physical and social functioning, and demographics. The OAA targets older adults with the greatest social and economic need. Older adults who receive in-home services (home-delivered meals, case management, and homemaker services) are often the ones with the highest level of need. For example, between one-half and three-quarters of in-home service clients live alone, more than half report mobility limitations, and one-third report limitations in 3+ activities of daily living (ADLs). In 2022, a module on emergency preparedness was added to the 16th NSOAAP. Findings show that one-third of in-home service clients experienced severe weather events such as a tornado, hurricane, or wildfire. Living alone and having medical devices that require electricity can be an added complication during emergencies. Among OAA clients, 41% of home-delivered meal, 60% of case management, and 42% homemaker clients reported having 3+ ADLs and rely on medical devices that require electricity. This presentation will discuss uses of the annual survey, findings from the rotating modules, and how researchers can access the data.

